# Supplementary material for: The temperature of emotions
Source: PLoS One. 2021 Jun 3;16(6):e0252408. doi: 10.1371/journal.pone.0252408 (PMC8174739; doi:10.1371/journal.pone.0252408)
Supplement: S1 File — (PDF) [file pone.0252408.s001.pdf]

S1 Table. Number of participants per first language and country of residence in Experiment 1

	English	Spanish	Japanese	Chinese
Argentina	-	4	-	-
Australia	1	-	1	2
Belgium	-	-	-	1
Bolivia	-	2	-	-
Canada	1	-	-	12
Chile	-	5	-	-
China	-	-	-	20
Colombia	-	7	-	-
Costa Rica	-	14	-	-
Denmark	-	2	-	7
Estonia	-	-	-	1
France	-	-	1	-
Germany	-	-	1	1
Guatemala	-	1	-	-
Honduras	-	1	-	-
Ireland	-	-	1	-
Italy	-	1	-	-
Japan	-	-	63	-
Mexico	-	37	-	-
New Zealand	2	-	2	1
Norway	-	4	-	3
Peru	-	1	-	-
Spain	-	8	-	1
Switzerland	-	-	-	2
Taiwan	-	-	-	1
United Kingdom	49	3	6	17
United States	101	4	4	7
Total	154	94	79	76

S1 Fig. Emotion adjectives in the four languages used in Experiment 1

A) English

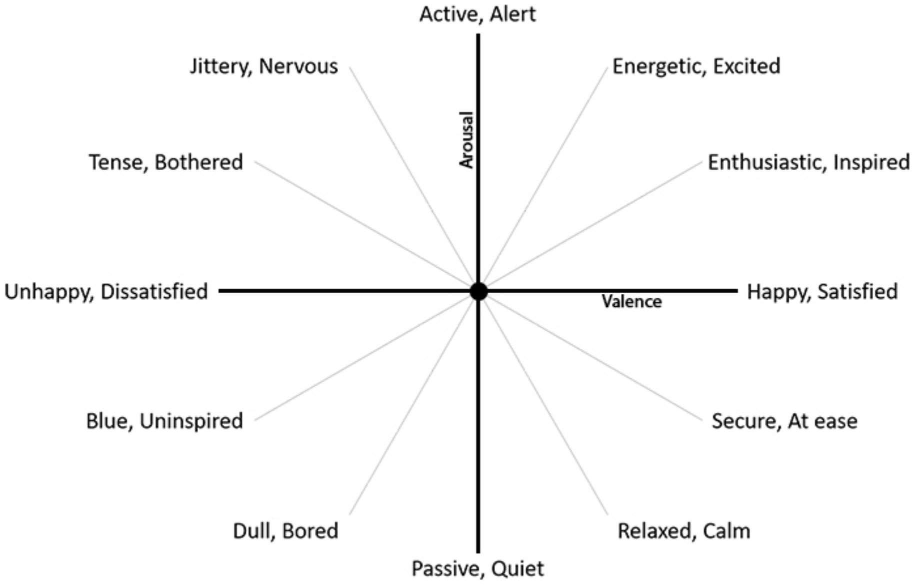

B) Spanish

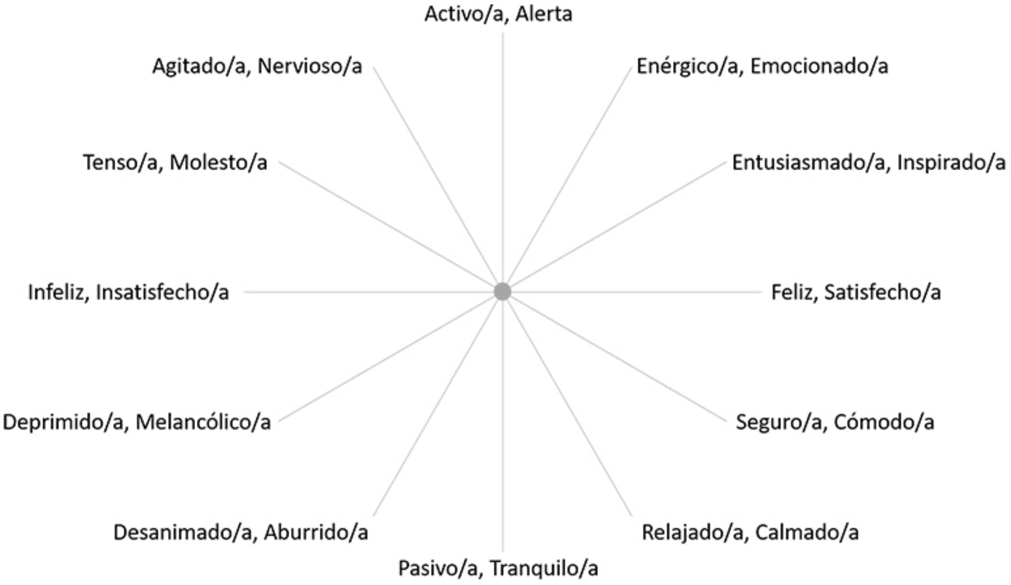

C) Japanese

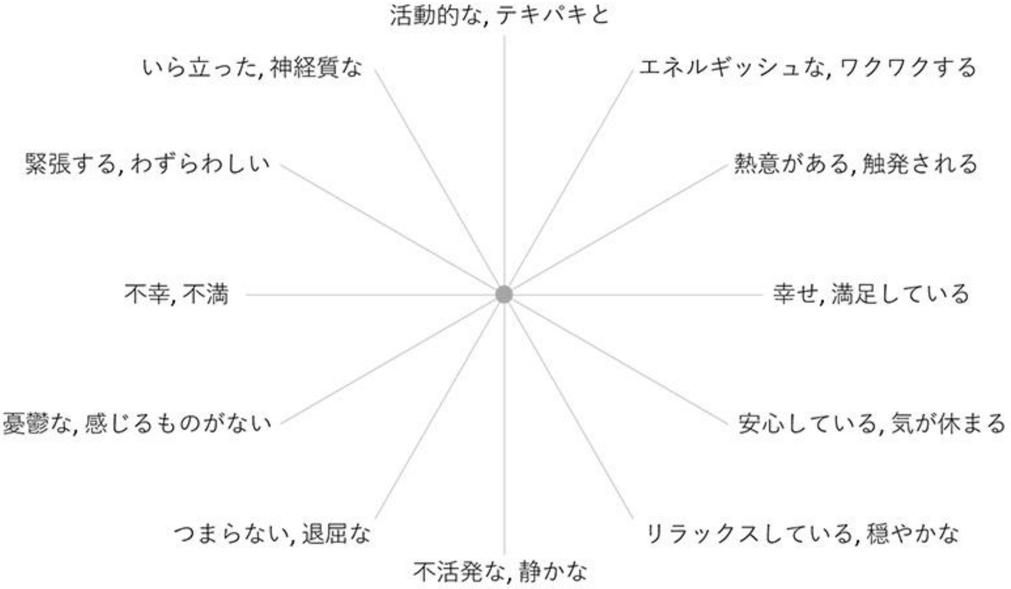

D) Chinese

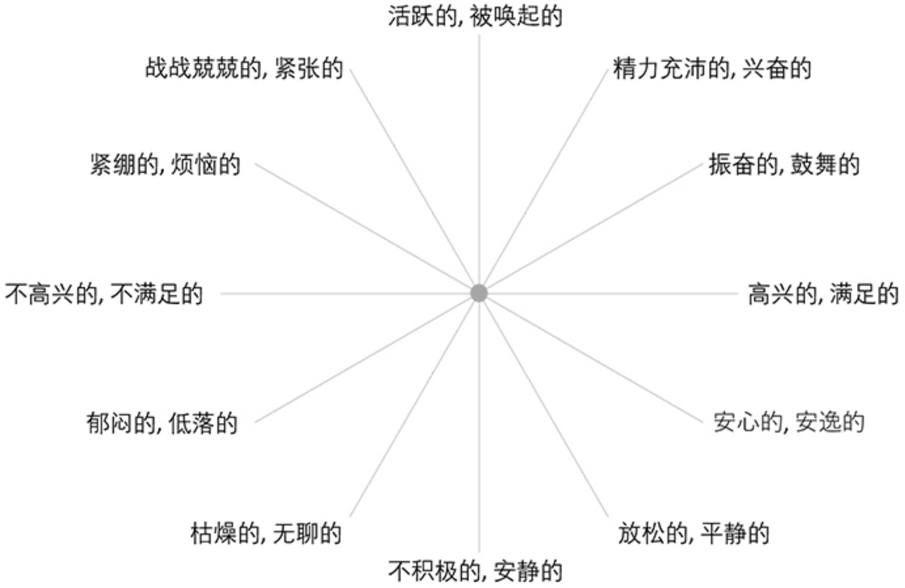

S2 Table. Varimax-rotated component matrix in Experiment 1.

	PC1	PC2
Temperature: 0 °C	0.509	0.246
Temperature: 10 °C	0.508	-0.178
Temperature: 20 °C	-0.178	-0.803
Temperature: 30 °C	-0.524	-0.070
Temperature: 40 °C	-0.422	0.508

S3 Table. Main and Interaction Relative Treatment Effects in Experiment 1

**A) Temperature: 0 °C**		English	Spanish	Japanese	Chinese
		0.49	0.48	0.53	0.51
Tense, Bothered	0.50	0.45	0.46	0.57	0.51
Jittery, Nervous	0.47	0.46	0.42	0.45	0.56
Active, Alert	0.36	0.33	0.37	0.34	0.41
Energetic, Excited	0.35	0.32	0.31	0.33	0.42
Enthusiastic, Inspired	0.35	0.32	0.35	0.33	0.41
Happy, Satisfied	0.37	0.35	0.35	0.34	0.43
Secure, At ease	0.43	0.41	0.40	0.43	0.48
Relaxed, Calm	0.50	0.52	0.47	0.43	0.57
Passive, Quiet	0.66	0.64	0.55	0.78	0.66
Dull, Bored	0.66	0.69	0.64	0.66	0.64
Blue, Uninspired	0.74	0.77	0.75	0.73	0.70
Unhappy, Dissatisfied	0.65	0.68	0.65	0.70	0.57

**B) Temperature: 10 °C**		English	Spanish	Japanese	Chinese
		0.47	0.48	0.56	0.53
Tense, Bothered	0.44	0.34	0.36	0.57	0.49
Jittery, Nervous	0.43	0.38	0.38	0.45	0.52
Active, Alert	0.41	0.33	0.44	0.40	0.47
Energetic, Excited	0.36	0.31	0.37	0.33	0.44
Enthusiastic, Inspired	0.37	0.32	0.38	0.33	0.46
Happy, Satisfied	0.45	0.38	0.45	0.44	0.52
Secure, At ease	0.52	0.46	0.48	0.57	0.58
Relaxed, Calm	0.59	0.57	0.59	0.56	0.65
Passive, Quiet	0.67	0.66	0.59	0.74	0.69
Dull, Bored	0.63	0.66	0.58	0.67	0.60
Blue, Uninspired	0.67	0.68	0.66	0.67	0.69
Unhappy, Dissatisfied	0.55	0.53	0.51	0.63	0.54

**C) Temperature: 20 °C**		English	Spanish	Japanese	Chinese
		0.45	0.52	0.57	0.50
Tense, Bothered	0.37	0.30	0.36	0.37	0.44
Jittery, Nervous	0.37	0.34	0.39	0.34	0.40
Active, Alert	0.60	0.50	0.60	0.63	0.65
Energetic, Excited	0.54	0.44	0.56	0.51	0.64
Enthusiastic, Inspired	0.55	0.48	0.62	0.48	0.63
Happy, Satisfied	0.67	0.60	0.70	0.68	0.70
Secure, At ease	0.71	0.62	0.70	0.73	0.78
Relaxed, Calm	0.69	0.61	0.70	0.72	0.75
Passive, Quiet	0.52	0.49	0.60	0.42	0.57
Dull, Bored	0.41	0.40	0.35	0.43	0.44
Blue, Uninspired	0.37	0.35	0.33	0.37	0.43
Unhappy, Dissatisfied	0.34	0.31	0.31	0.31	0.42

**D) Temperature: 30 °C**		English	Spanish	Japanese	Chinese
		0.48	0.53	0.52	0.49
Tense, Bothered	0.53	0.53	0.59	0.50	0.51
Jittery, Nervous	0.55	0.51	0.60	0.62	0.49
Active, Alert	0.65	0.63	0.66	0.62	0.68
Energetic, Excited	0.69	0.68	0.72	0.69	0.68
Enthusiastic, Inspired	0.65	0.66	0.61	0.68	0.66
Happy, Satisfied	0.63	0.65	0.65	0.56	0.64
Secure, At ease	0.51	0.53	0.59	0.41	0.51
Relaxed, Calm	0.46	0.44	0.50	0.41	0.49
Passive, Quiet	0.34	0.30	0.39	0.28	0.37
Dull, Bored	0.35	0.28	0.37	0.34	0.39
Blue, Uninspired	0.28	0.23	0.27	0.31	0.32
Unhappy, Dissatisfied	0.39	0.34	0.36	0.42	0.45

**E) Temperature: 40 °C**		English	Spanish	Japanese	Chinese
		0.51	0.50	0.48	0.49
Tense, Bothered	0.63	0.69	0.71	0.55	0.57
Jittery, Nervous	0.62	0.61	0.64	0.73	0.52
Active, Alert	0.59	0.65	0.58	0.56	0.58
Energetic, Excited	0.65	0.71	0.62	0.68	0.60
Enthusiastic, Inspired	0.62	0.65	0.54	0.70	0.60
Happy, Satisfied	0.51	0.53	0.50	0.46	0.53
Secure, At ease	0.38	0.43	0.40	0.30	0.39
Relaxed, Calm	0.34	0.35	0.37	0.31	0.35
Passive, Quiet	0.35	0.34	0.35	0.34	0.38
Dull, Bored	0.40	0.35	0.45	0.35	0.44
Blue, Uninspired	0.36	0.33	0.36	0.38	0.37
Unhappy, Dissatisfied	0.51	0.48	0.50	0.57	0.50

Relative treatment effects (RTEs) indicate the tendency of participants to have higher (or lower) ratings of associations between an emotion and a given temperature compared to all the participants' ratings for the other emotions. RTEs range from 0 to 1. Larger differences between RTEs indicate larger differences in association ratings.
