# Supplementary material for: The temperature of emotions
Source: PLoS One. 2021 Jun 3;16(6):e0252408. doi: 10.1371/journal.pone.0252408 (PMC8174739; doi:10.1371/journal.pone.0252408)
Supplement: Attachment — Submitted filename: Response to Reviewers.pdf [file pone.0252408.s002.pdf]

Response to reviewers: The temperature of emotions

We thank the editor for the opportunity to resubmit our manuscript and the reviewers for their insightful comments. We have revised our paper according to their suggestions; below we address each one of them. Reviewers' comments are in italics, and our responses are in regular font. All the additions are in blue font and the deletions are in red font in the *Manuscript with Track Changes*.

Reviewer #1

The authors report on 2 studies investigating implicit and explicit associations between temperatures and emotion adjectives. The authors find that cold temperatures are associated with low arousal negative emotions and hot temperatures are associated with high arousal positive emotions (though slightly also with high arousal negative emotions in Study 1). Results are largely consistent across first languages.

These are interesting studies in fairly well-powered samples that advance our understanding of emotion-temperature associations. Overall, I think this paper makes a nice next step in the literature. That said, I have some concerns about the analytic approaches and the way the results are integrated with prior literature. I outline these below.

> We are grateful to the reviewer for the thorough and useful comments. We have carefully considered all the suggestions in our revision, and we have improved the analysis in the manuscript.

Major concerns

Comment 1: *Background literature that is closely related to the current study is not clearly described. The reader should get a few more details on citations 9-16, the Bergman et al. citation [58], and Bruno et al. [1] (i.e., what do you mean by "emotionally warm."). Because the methods and directions of the results of these papers are not very clearly described, the reader is left unsure of what exactly the prior literature tells us and what is novel about the current study. I came to understand this in time in the paper, but it should be described more clearly earlier on in the introduction.*

> We have now more clearly described the relevant background literature. We have elaborated on the findings, methods, and directions of the cited studies (see lines 50-91, 99-105, and 209-214 in the manuscript).

Comment 2: *I found the paper's current reviews of the embodied cognition and evolutionary psych literatures either incomplete or confusing. There's much more work than just Inagaki's research on embodied processes, and the evolutionary psych paragraph just references an MRI study that I don't think actually supports the authors' argument… I actually feel like much of this paper's results could more elegantly and succinctly summarized by just grounding it in the constructionist*

theory of emotion (see reviews by Barrett, 2017, 2006, as well as relevant papers by Lindquist, Satpute, etc.). This framework suggests that emotion concepts are used to parse core affect into specific emotion types, and it makes sense that temperature would feed into emotion concepts. Sievers et al. also have a recent paper in PNAS that could help with this multimodal representation of affect/emotion.

We thank the reviewer for the insights on the theoretical background for our manuscript. Following this comment, we have more parsimoniously framed our study on the theory of constructed emotion following the suggested literature. We have used the studies on homeostatic processes in the original manuscript to complement and exemplify the constructionist theory and provide a holistic view (see lines 143-144 and 170-178 in the manuscript).

Comment 3*: At times the authors make claims like "emotions have meanings and are experienced differently based on the concepts with which they are associated" and "there is a high degree of universality in the experience of emotions," but these claims are a huge topic of debate. The first is a tenet of constructionist theory and the second is a claim of the basic emotion theory. I'd either remove or phrase claims like these more speculatively. It also seems unclear what the authors' stance is when these opposing claims are both held in one paper.*

We have removed the claims on the universality of emotions and provided a more nuanced discussion. We discuss the large similarity in the associations across languages building on the common semantic framework based on valence-arousal and homeostatic processes as suggested by Jackson et al. [1]. We believe our stance on the constructionist theory of emotions is now clearer (see lines 826-861 in the manuscript).

Comment 4*: One methodological concern is that recruiting through professional networks could introduce bias? Was this controlled for? What were the Ns for different recruitment types?*

The recruitment process outside dedicated recruitment platforms (i.e., Prolific, Lancers) took place via social media posts in the authors' academic and professional networks, which include people from different demographic, academic and professional backgrounds, as well as age. It should be noted that those from professional networks were not professional researchers but business school students and alumni. We have made this clearer, and we have detailed the number of people recruited through each method (see lines 337-342 in the manuscript). Taking full consideration of the comment, we have explored controlling for the recruitment platform in the Linear Mixed Model detailed below (see the footnote from line 522, on page 19).

Comment 5*: Similarly, is there any assurance that different language groups are not confounded by other variables? The age differences are pronounced. Could you test for significant differences across these? Do results vary when this is controlled for?*

We attempted to mitigate potential confounding factors by retaining only speakers of the native languages studied. However, we cannot fully rule out their effect as we state in lines 571-573. In full consideration of this and other comments from the reviewer, we conducted an additional analysis fitting a Linear Mixed Model (LMM) controlling for age and gender (more details are provided in the response to Comment #8). The results of the LMM did not differ from the original ones.

Comment 6*: I found it odd that the study design involved presenting PAIRS of emotion words. How do we know that (some) readers aren't just reading the leftmost word (esp. in study 2 when speed is critical)? This is a methodological detail that should be highlighted earlier on for readers and noted as a limitation. I think it makes interpretation less clear cut.*

We appreciate this reflection. While we cannot know with absolute certainty whether some participants read the whole text or not, the use of the pairs of emotion words has been validated in multiple studies [2–4]. Nevertheless, we mentioned this potential limitation in the Limitations and Future Directions section (see line 900-904 in the manuscript).

Comment 7*: I'm not sure I follow the authors' PCA analysis. Specifically, I think it's odd to add in valence/arousal values, especially when these values are essentially made up by the authors. Are there any normative data that they could use instead? Or instead, I would just encourage the authors to run the PCA on the temperature analyses and then show that the two axes essentially line up with valence and arousal. Right now, they make interpretations of how "valence was positively related to ambient temperature (20 °C)" and that "valence was related to ambient or comfortable temperature concepts and arousal was related to higher temperatures," but I don't think these interpretations are really logical conclusions from PCA… The authors essentially just made up the valence/arousal values, and factors are only associated with certain variables after controlling for other factors… I like the idea of applying PCA, but I don't think the way they're executing/interpreting it is very strong.*

We thank the reviewer for the valuable comment. We have removed the emotion words' valence and arousal values and conducted the PCA only with the temperature categories. We have also modified the interpretation of the PCA and discussed the alignment of the valence and arousal axes, as well as the direction of the associations in the circumplex with the change in temperature (see lines 459-481 in the manuscript).

Comment 8*: In fact, my interpretation of the Fig 4 plot is that there is nice evidence that different temperatures are essentially associated with different areas of the circumplex (the "peak" of the wave of emotion associations moves from far right to the middle then to the left of each plot as temperature increases, suggesting that people are essentially moving counterclockwise through the circumplex starting in the low left and ending in the upper middle as temp increases). Is there some way to rethink the analytic plan so that temperature is treated as a continuous variable, rather than a series of independent measures? I can't think of a way right now, but perhaps*

consulting with a statistician could reveal some kind of interesting multidimensional scaling approach?

We thank the reviewer for this insightful suggestion. We have added an additional analysis, in which we transformed the temperature to a continuous variable and fit a Linear Mixed Model (LMM) with temperature as a continuous variable, the interaction between emotion and language as fixed factors, age and gender as covariates, and subject ID as random factor. The analysis allowed us to account for the idiosyncratic variability of participants and to show more clearly the pattern of associations in the emotion circumplex as temperature increased. In addition, relevant to Comment #5, we controlled for age and gender (see lines 423-437 and 514-533 in the manuscript).

Comment 9*: I also feel like greater statistical expertise might be needed to think through the language analysis… Right now, it seems like the authors correlate matrices that are just average ratings across all speakers of each language… This sucks out all variance within each language, a suboptimal approach. Is there some way to do a nested analysis? Perhaps not but thinking this through would be helpful.*

We thank the reviewer for this comment. We agree that this analysis overlooks the variance within each language. Nevertheless, our objective with this analysis consisted of uncovering overall similarity patterns in the emotion-temperature associations across languages and not within them. Furthermore, our relatively small sample size within each language category (76-154) made it difficult to justify focusing on variances within each category. To this end, we conducted a similar analysis to that of Jonauskaite's et al. [5] in their large color-emotion associations across languages study. We have cited this article in our manuscript (see lines 438-442 in the manuscript).

Comment 10*: I'm glad the authors point out the important limitation that people may have thought of "hot" and "cold" as referring to affective states, not temperatures. Perhaps a future direction would be to use images or phrases associated with hot things in the IAT (e.g., press left if you see a picture of a warm scene/activity or a picture of a positive facial expression. Press right if you see a picture of a cold scene/activity or a negative facial expression).*

We agree with the reviewer and consider this is an interesting avenue for future research. We have included these points in the Limitations and Future Directions section (see lines 898-900 in the manuscript).

Smaller concerns

Comment 11*: I'm not sure about the phrase "temperature concepts." Do humans really have a specific concept around "20 degrees Celsius?"*

We thank the reviewer for this comment. We believe the phrase temperature concepts is appropriate, as we are not studying physical temperature but the notions of different

temperatures. Moreover, research has shown that exposure and familiarity with different temperatures can change how they are perceived [6].

Comment 12*: I found myself tripping up with interpretation phrases like "participants associated the emotions Enthusiastic/Inspired and Passive/Quiet with 20°C." It seems like the authors are basing these on the ANOVA results showing that average ratings for the 20°C question are higher for these emotions than others. But just because these ratings are higher for these emotions than others doesn't really mean that they "significantly associate" these concepts… I feel like two adjustments are needed here: 1) when talking about average ratings in the task just refer to them as "average ratings were higher for X emotion than Y emotion" rather than saying that there were "significant associations" and 2) I might stick to just talking about how temperatures showed "peaks" around certain parts of the circumplex rather than overinterpreting which emotions were higher than all the others.*

We thank the reviewer for this comment. We have made the suggested adjustments and discussed the results from the perspective of the counterclockwise movement around the emotion circumplex starting in the lower left side as temperature increases from 0 °C to 40 °C throughout the manuscript (see lines 445-455, 473-481, 493-513, and 747- in the manuscript).

Comment 13*: In abstract, please specify the Ns of study and clarify which language is studied in Study 2*

We have added these details in the abstract (see lines 17-18 and 22 in the manuscript).

Comment 14*: In intro, maybe use the phrase "chilled with fear" instead of "cold fear"? I don't know if I've ever heard the second in common parlance.*

We have made the suggested change (see lines 30-31 in the manuscript).

Comment 15*: How exactly does "this set of emotion words mitigate the risk of vague emotions and poor usability?"*

We have added more details on the advantages of these emotion words. More specifically we point at its validation in in multiple product-oriented studies and consequently its applicability to multiple fields (see lines 369-371 in the manuscript).

Comment 16*: Could Fig 2 include the average ratings in each cell of the table?*

We have added the average ratings in the heat map.

***Comment 17**: I think the phrase "on the other hand" is misused in the paper. It is used as a phrase for "additionally" when I think it usually implies "for an opposing perspective."*

We have corrected the use of the phrase throughout the manuscript.

***Comment 18**: I couldn't follow the authors' evolutionary argument about blushing. Why is greater heat loss in these areas adaptive? How this gets at the conceptual overlap between temperature and emotion that the authors are studying?*

The description of blushing exemplifies the homeostatic process in response to physiological changes caused by certain emotional experiences. This ties to the situated conceptualizations of emotional experiences and bodily temperature changes that occur in tandem (see lines 198-200 in the manuscript).

<u>Reviewer #2</u>

Dear Mr. Escobar, I have had the opportunity to review the authors' submission, "Temperature of Emotions". This paper investigates whether temperatures are systematically and specifically associated with emotions. The authors conduct two studies to assess this relationship: In study 1, individuals in four cultures, evaluated 12 emotion terms (sampled from around the circumplex) on five temperatures (ranging every 10 degrees C from 0 to 40). In study 2, an adapted version of the IAT was used to see whether (participants in English) showed implicit biases to happy (+ valence)/active (high arousal) and warm pairings, and unhappy (- valence)/passive (low arousal) and cold pairings.

Overall, this paper is extremely well-written, with a solid introduction and review of the extant literature, as well as sound in design and analyses. The results are straight-forward, aligned with the authors' hypotheses, and the implications for importance are well-stated. I was also impressed by the detail to design (counterbalancing in both studies, use of the SAM in study 1, and the statistical presentation (CIs, comparison of effects sizes). Figures were nicely displayed, and all statistics conformed to the latest recommended APA standards and were thoughtfully conducted.

We thank the reviewer for her valuable insights and positive evaluation. We have addressed all the comments below and made the corresponding modifications in the manuscript.

***Comment 1**: The only criticism I have is one already noted by the authors. That is, the collection of reaction time on individual devices, which vary in speed, etc. Although the study is within-subjects (helping to reduce differences among platforms/computer speeds), I still find it concerning to rely on differences of < 200 ms as statistically different when reported from uncontrolled/standardized devices. There is not much to be done, at this point, but perhaps a replication in the lab is possible in the near future (I am assuming in person data collection was limited as a result of the COVID pandemic).*

Thank you for pointing this out. Gorilla has been shown to be highly accurate and effective in the presentation of visual stimuli with variability under 6 ms inter-trial variability in the most common browsers (Firefox, Safari, Edge) and sub-millisecond variability in Chrome and Ubuntu [7,8]. In fact, Gorilla, along with PsychoPy, have been shown the best performers. We elaborate on this in the manuscript (see lines 645-654).

I find the overall subject to be of interest and timely, and of appropriate content for this journal. It not usual that I have few to no concerns of criticisms before publication. As it stands, I only have a few clarifying points.

Minor criticisms

Comment 2*: It would be interesting to know in Study 1 the location (country) from which participants originated or currently lived. This might help disentangle cultural from linguistic effects. Relatedly, in Study 2, it would be nice to know whether English-speaking participants (all living in the UK) were familiar with another language.*

We thank the reviewer for this comment. The table with the breakdown of the current country of residence of participants per language is in the Supplementary Materials (Table S1). We made this clearer in the manuscript (see lines 353-355 in the manuscript) Regarding Study 2, two participants indicated they were native speakers of another language in addition to English. One of them was also a native speaker of French and the other one of Serbian. We have added these details in the manuscript (see lines 592-593 in the manuscript). We believe future studies investigating these effects on multilingual or multicultural people is an interesting path of further research

Comment 3*: I am not sure how ATS statistics (used through R) are similar/different to the Wilcoxon non-parametric signed-based statistics. It would be helpful to make a comparison to these more "common" statistics. There seems to be some debate as to whether homogeneity of variances is required for the Wilcoxon (which you say you do not have).*

We have added the most important advantages of the ATS over more traditional methods, namely effectiveness robustness in dealing with heteroscedastic data and factorial designs [9,10], as well as better performance than Wald-type statistic [11] (see lines 416-420 in the manuscript).

Comment 4*: Although the results for Study 1 are clear and presented both across languages/cultures and for each separately, I would like to see some discussion (even if speculative) as to why Chinese and Spanish showed higher correlation than presumably between Chinese and Japanese (because it is not listed as higher or lower). To that end, it would also be interesting to expound on why there are specific some cultural/linguistic differences (albeit minimal to overwhelming consistent pattern across languages).*

We thank the reviewer for this insightful comment. We have added a short discussion on the small differences in the associations across languages related to linguistic discrepancies and meanings of emotions in different languages [1], differences in the encoding of emotions in languages [12], and the effect of temperature on the subjective experience of affective stimuli [6]. Regarding the higher correlation between Chinese and Spanish vs. Chinese and Japanese, we speculate this can be a result of more international cultural exposure (see lines 845-861 in the manuscript).

Comment 5*: I would appreciate a further discussion of the improved scoring methods of the d statistic of Greenwald and colleagues. I do not follow the use of replacing the error trials with the mean RT + 2 SDs.*

The treatment of error rates is highly recommended as they contain important information about participants, it increases statistical power, and it improves the accuracy and validity of the analysis [13]. We have elaborated on the benefits of the treatment of errors in the manuscript (see lines 670-673 in the manuscript).

Overall, I believe that the research is potentially interesting and worthy of publication.

References

1. Jackson JC, Watts J, Henry TR, List J-M, Forkel R, Mucha PJ, et al. Emotion semantics show both cultural variation and universal structure. Science (80-). 2019;366: 1517–1522. doi:10.1126/science.aaw8160

2. Jaeger SR, Roigard CM, Jin D, Xia Y, Zhong F, Hedderley DI. A single-response emotion word questionnaire for measuring product-related emotional associations inspired by a circumplex model of core affect: Method characterisation with an applied focus. Food Qual Prefer. 2020;83: 103805. doi:10.1016/j.foodqual.2019.103805

3. Jaeger SR, Roigard CM, Chheang SL. The valence × arousal circumplex-inspired emotion questionnaire (CEQ): Effect of response format and question layout. Food Qual Prefer. 2021;90: 104172. doi:10.1016/j.foodqual.2020.104172

4. Jaeger SR, Xia YX, Le Blond M, Beresford MK, Hedderley DI, Cardello A V. Supplementing hedonic and sensory consumer research on beer with cognitive and emotional measures, and additional insights via consumer segmentation. Food Qual Prefer. 2019;73: 117–134. doi:10.1016/j.foodqual.2018.11.015

5. Jonauskaite D, Abu-Akel A, Dael N, Oberfeld D, Abdel-Khalek AM, Al-Rasheed AS, et al. Universal patterns in color-emotion associations are further shaped by linguistic and geographic proximity. Psychol Sci. 2020; 1–16. doi:10.1177/0956797620948810

6. Parker PM, Tavassoli NT. Homeostasis and consumer behavior across cultures. Int J Res Mark. 2000;17: 33–53. doi:10.1016/S0167-8116(00)00006-9

7. Anwyl-irvine A, Dalmaijer ES, Hodges N, Evershed JK. Realistic precision and accuracy

of online experiment platforms, web browsers, and devices. Behav Res Methods. 2020. doi:10.3758/s13428-020-01501-5

8. Bridges D, Pitiot A, Macaskill MR, Peirce JW. The timing mega-study: Comparing a range of experiment generators, both lab-based and online. PeerJ. 2020;8: e9414. doi:10.7717/peerj.9414

9. Erceg-Hurn DM, Mirosevich VM. Modern robust statistical methods: An easy way to maximize the accuracy and power of your research. Am Psychol. 2008;63: 591–601. doi:10.1037/0003-066X.63.7.591

10. Zimmerman DW. Statistical significance levels of nonparametric tests biased by heterogeneous variances of treatment groups. J Gen Psychol. 2000;127: 354–364. doi:10.1080/00221300009598589

11. Bathke AC, Schabenberger O, Tobias RD, Madden L V. Greenhouse-Geisser adjustment and the ANOVA-type statistic: Cousins or twins? Am Stat. 2009;63: 239–246. doi:10.1198/tast.2009.08187

12. Lindquist KA. The role of language in emotion: Existing evidence and future directions. Curr Opin Psychol. 2017;17: 135–139. doi:10.1016/j.copsyc.2017.07.006

13. Richetin J, Costantini G, Perugini M, Schönbrodt F. Should we stop looking for a better scoring algorithm for handling implicit association test data? Test of the role of errors, extreme latencies treatment, scoring formula, and practice trials on reliability and validity. PLoS One. 2015;10: 1–23. doi:10.1371/journal.pone.0129601
