# Supplementary material for: The temperature of emotions
Source: PLoS One. 2021 Jun 3;16(6):e0252408. doi: 10.1371/journal.pone.0252408 (PMC8174739; doi:10.1371/journal.pone.0252408)
Supplement: Attachment — Submitted filename: The temperature of emotions_response to reviewers.pdf [file pone.0252408.s003.pdf]

Response to reviewers: The temperature of emotions

We thank the editor for the opportunity to submit a revised version of our manuscript and the reviewers for their new comments. We have incorporated all their suggestions in the paper. Below we address each comment. Reviewers' comments are in italics, and our responses are in regular font. All the additions are in blue font and the deletions are in red font in the *Manuscript with Track Changes*.

Reviewer #1

The authors have done a great job incorporating edits and the manuscript is much improved. I just have a few small points on the revision that I think will improve interpretability.

We are glad that the reviewer found our edits satisfactory.

Comment 1*: PCA results are interesting. Dim 1 seems to be arousal and Dim 2 is valence. This is opposite most findings (Dim1 = valence, Dim 2 = arousal), but it sometimes happens depending on the stimuli included. But I think it needs to be narrated for readers a bit. Perhaps labeling the axes, adding notes in the main text, and even labeling the quadrants would be helpful. I also don't think you need to interpret 20 degrees "aligning" w the vertical axis and 30 degrees "aligning" w horizontal axis (alignments are weak and not quite sure how to interpret them…). Finally, the rotation is described as counterclockwise around the circumplex, but doesn't increasing temp move clockwise on the PCA projection? In the discussion I agree that it moves counterclockwise in a "canonical" circumplex, but the differences between what is found canonically and what is presented in the figure are never clarified for readers.*

We thank the reviewer for this reflection. Based on their suggestion, we have edited the results of the PCA to improve their interpretability. In particular, we have removed the interpretation of the alignment of the axes (lines 409–411). We have also more clearly explained the corresponding dimensions and the clockwise movement in the PCA projection (lines 412–415), and we explained the difference with respect to the canonical emotion circumplex (416–422). In addition, we added a few lines in the notes of the PCA plot (Fig 3). We have also clarified when we refer to the canonical circumplex model of affect in the discussion and throughout the text to avoid confusion.

Comment 2*: The LMM analysis is v important and interesting. I think the results could be more fully discussed. In particular, there are some interesting differences between languages that are not explored at all. There's a few sentences about it in the general discussion, but talking through a few of the findings in the results of Study 1 seems merited.*

We thank the reviewer for this comment. Following the reviewer's suggestion, we have added a short discussion of the LMM results in terms of the differences across languages. We have added a few lines in the Results of Experiment 1 regarding the lower temperature ratings of the Chinese speaking participants for the high arousal emotions, especially the

negative valence ones. We have also described the large heterogeneity in two specific emotion categories (lines 466–473 and 773–775 in the manuscript).

Reviewer #2

Thank you for your revision on the ms: "The temperature of emotions". I found that it addressed my initial concerns and those brought up by the other reviewers. I found myself actually agreeing with many of the initial points of the other reviewers, despite my very positive initial review. To that end, you have not only acknowledged and sufficiently addressed my concerns, but you have also addressed their concerns (to my satisfaction, although of course they may disagree).

We thank the reviewer for her comments, and we are glad we successfully addressed the reviewers' comments.

***Comment 1**: The one point that I did not mention initially and would like to strongly recommend you elaborate upon even a bit more, is the theoretical grounding of Psychological Constructionism. In fact, I have published on this work extensively, and have a color-emotion paper that uses the Theory of Constructed Emotions (Barrett, 2006, 2019) to explicitly test whether colors and emotions pairings are both specific and consistent (as that theory would expect). I believe that you would find that paper informative and perhaps some of the language within the rationale might even help you frame your comment to reviewer 1, comment 2.*

Beyond that, I am happy and satisfied with the revisions. I will recommend to the editor that your submission be published.

References: Fugate, J. M. B., & Franco, C. L. (2019). What color is your anger? Assessing color-emotion pairings in English speakers. Frontiers in psychology, 10, 206.

We thank the reviewer for the comment and her recommendation. Taking full consideration of this comment, we have added some new lines in the framing of our paper based on the constructionist theory with insights from the referenced paper (see lines 149–152).
